# Comparative Effectiveness of Three Myopia Control Lenses: A Multicenter, Real-World Study of 5182 Adolescents and Children in Chongqing, China

**DOI:** 10.1167/tvst.15.5.22

**Published:** 2026-05-27

**Authors:** Xiao Chen, Haonan Pan, Jing Yi, Yuanyuan Tang, Jing Rao, Shiwei Ren, Quanlan Li, Jia Guo, Andrea Llovet-Rausell, Chunmei Chen, Qizhi Zhou, Yi Wang

**Affiliations:** 1 Aier Eye Hospital, Jinan University, Guangzhou, People's Republic of China; 2 Aier Eye Hospital Group Company Limited, Changsha, People's Republic of China; 3 Department of Health Statistics, School of Public Health, Chongqing Medical University, Chongqing, People's Republic of China; 4 Chongqing Eye and Vision Care Hospital, Aier Eye Hospital Group, Chongqing, People's Republic of China; 5 Chongqing Aier Children's Eye Hospital, Chongqing, People's Republic of China; 6 Chongqing Aier Eye Hospital, Chongqing, People's Republic of China; 7 Inspur Enterprise Cloud Technology Co., Ltd., Shandong, People's Republic of China; 8 Clínica Baviera - Aier Eye Hospital Group, Valencia, Spain; 9 Chongqing Aier - Mega Eye Hospital, Chongqing, People's Republic of China

**Keywords:** real world, myopia control, myopia progression, myopia control lenses, orthokeratology (OK)

## Abstract

**Purpose:**

The purpose of this study was to assess the 1-year myopia control efficacy of 3 optical interventions—lenslet-based design (LD) spectacle lenses, peripheral defocus (PD) spectacle lenses, and orthokeratology (OK) contact lenses—in real-world clinical settings among children and adolescents in China.

**Methods:**

In this multicenter retrospective study, we analyzed the medical records of 5182 participants aged between 6 and 16 years old. The participants were divided into four groups according to the myopia control method. The axial length (AL) and spherical equivalent refraction (SER) of the right eye were used to assess myopia progression. Myopia progression was compared across the matched cohorts using propensity score matching and multiple linear regression. Subgroup analyses were used to assess the differential efficacy of lenses, stratified by age, sex, and baseline refractive status.

**Results:**

At the 1-year follow-up, the LD group demonstrated SER progression of −0.23 ± 0.09 diopter (D) and 0.12 ± 0.06 mm of axial elongation (AE) compared with the single vision (SV) group. The OK group demonstrated an AE reduction of 0.13 ± 0.06 mm compared with the SV group. Stratified analyses demonstrated consistent superiority of OK lenses over PD and SV lenses across all subgroups (*P* < 0.05 for AL), whereas LD and OK lenses showed comparable efficacy in AL control (*P* > 0.05).

**Conclusions:**

LD and OK lenses demonstrated superior efficacy in controlling myopia compared with PD and SV lenses, with comparable AL control between the two interventions.

**Translational Relevance:**

This large-scale, real-world evidence translates the efficacy of myopia-control lenses into actionable guidance for pediatric clinical practice.

## Introduction

Cases of myopia have been on the rise globally over the past 3 decades.^1^ In China, the prevalence of myopia is currently as high as 71.34% in certain provinces.^2^ Moreover, in recent research projections, it has been estimated that the prevalence of overall myopia and high myopia will reach 61.3% and 17.6%, respectively, by 2050.^3^ Myopia is closely associated with the development of numerous eye conditions. The risk of myopia-related macular degeneration, retinal detachment, cataracts, open-angle glaucoma, and blindness increases as myopia progresses.^4^^,^^5^ These alarming trends highlight the urgent need for effective myopia control interventions during childhood and adolescence.

Optical interventions, particularly orthokeratology (OK) contact lenses, lenslet-based design (LD) spectacle lenses—such as defocus incorporated multiple segments (DIMS) lenses—and peripheral defocus (PD) spectacle lenses—such as Myovision—are widely used in China to control myopia. Current evidence predominantly derives from randomized controlled trials (RCTs).^6^^–^^8^ Although RCTs are considered the gold standard for assessing efficacy, their strict inclusion and exclusion criteria limit generalizability to real-world clinical populations. In routine practice, heterogeneous baseline characteristics, variable treatment adherence, and challenges in recruiting treatment-naïve participants may introduce bias.^9^ Thus, real-world data and real-world evidence (RWE) play a crucial role in complementing RCT findings by providing insights into the effectiveness of interventions across broader, unselected populations.^10^

At present, large-scale, real-world studies (RWSs) that directly compare the efficacy of multiple types of myopia control lenses remain limited. Existing RWSs are often constrained by small sample sizes (*n* = 77–317)^10^^–^^13^ or a narrow focus on refractive outcomes alone, without assessing axial length (AL) changes.^14^ For example, although their study was informative, Liu et al.^14^ evaluated only refractive changes to determine the efficacy of DIMS lenses and did not include AL measurements. This omission is important because AL elongation is a fundamental driver of myopia progression, contributing directly to refractive error development and inducing biomechanical stretching, retinal flattening, and peripheral hyperopic defocus—key mechanisms that increase the risk of sight-threatening complications.^15^

To address these gaps, in this RWS, we analyzed data from a large cohort across four major ophthalmic hospitals in Chongqing, China. We evaluated spherical equivalent refraction (SER) and AL changes with three types of myopia control lenses (OK, LD, and PD) compared with single-vision (SV) lenses. Propensity score matching (PSM) reduced confounding bias, thereby enhancing the validity of comparative effectiveness estimates.^16^ Our findings provide strong RWE to guide personalized lens selection across diverse pediatric populations.

## Methods

### Data Sources and Ophthalmic Examinations

The medical records of patients aged 6 to 16 years who were prescribed LD, PD, OK, or SV lenses at 4 Aier Eye Hospitals in Chongqing, China, between January 1, 2021, and February 18, 2024, were retrospectively reviewed. Extracted data included sex, age, examination dates, and prescription dates, lens parameters, SER, and AL. All records were extracted from the standardized electronic health record (EHR) system (Pengsheng, China). Low-dose atropine eye drops had not yet been approved by the China Food and Drug Administration for use in mainland China during the study follow-up period. All four hospitals followed the same clinical workflows for lens prescription. First, the patient's basic information was recorded, including age, sex, and ocular and systemic disease history. An ophthalmic examination was then conducted. Finally, a refractive examination was performed, and a lens prescription was issued. Refractive assessments were performed according to standard refraction protocols, with cycloplegic refraction using 0.5% cyclopentolate eye drops (Santen Pharmaceuticals, Osaka, Japan). Patients were instilled 3 times at 3 to 5-minute intervals, followed by subjective refraction 30 minutes after the last instillation. The final refractive prescription was determined according to subjective refraction measurements, adhering to the principle of maximum positive lens power for best-corrected visual acuity. To validate the reliability of subjective refraction, we compared the cycloplegic subjective refraction results with objective refraction values obtained as the average of three consecutive autorefractor measurements (ARK-1; NIDEK, Shanghai, China) under cycloplegia. A subset of data was randomly selected for a Bland-Altman analysis to assess the agreement between the two methods (Supplementary Fig. S1). AL was measured using an Optical Interferometric Biometer (AL-SCAN; Nidek Co., Aichi, Japan). Three consecutive measurements were obtained for each eye and averaged for analysis. SER progression was calculated as the difference between the final subjective refraction and baseline prescription. Axial elongation (AE) was defined as the difference between the final and baseline AL measurements. This study was conducted in accordance with the tenets of the Declaration of Helsinki and was approved by the Institutional Review Boards (IRBs) of the Aier Eye Hospital Group (AIER2023IRB088) and Chongqing Eye and Vision Care Hospital, Aier Eye Hospital Group (IRB 2024009). As the data were extracted from EHRs and de-identified, the IRB waived the requirement for patient informed consent.

### Inclusion Criteria

The inclusion criteria were as follows:

(1) Age at the time of lens prescription: Spectacle lenses (LD, PD, and SV): 6 to 16 years; OK lenses: 8 to 16 years.
(2) Interval between lens prescription and baseline examination: ≤30 days.
(3) Follow-up duration: 12 ± 2 months.^17^
(4) Baseline refractive criteria: SER: −0.50 to −5.875 diopter (D); cylinder: 0 to −1.50 D (to ensure a fair comparison among the 3 myopia control modalities [LD lenses, PD lenses, and OK lenses] in a uniform patient population).
(5) Best-corrected visual acuity ≥0.0 (LogMAR).
(6) Anisometropia: <2.00 D.

### Exclusion Criteria

The exclusion criteria were as follows:

(1) Concurrent use of other myopia control interventions (during the observation period, EHR demonstrated that individuals were simultaneously fitted with or alternately used two or more types of myopia control lenses).
(2) Special lens types: anti-fatigue, progressive or photochromic lenses, soft contact lenses, and rigid gas-permeable contact lenses.
(3) Patients without a lens prescription.

### Lens Types

Four study groups were defined as follows. (1) The LD group: this group comprised LD spectacle lenses, typically featuring a central optical zone for distance vision correction, with the peripheral zone incorporating densely distributed micro-lenses that induced myopic defocusing through high-intensity, spatially discrete optical signals (DIMS,^18^ highly aspherical lenslets [HAL],^19^ individualized ocular refraction customized [IORC] lenses,^20^ and other LD design types, forming a total of 4 subtypes). The other LD design types comprise six commercially available lenses, five based on spherical micro-lens arrays and one (ZEISS MyoCare with Cylindrical Annular Refractive Element technology) based on a micro-cylindrical array. (2) The PD group: this group involved PD spectacles (4 subtypes: YAGE PD, Shamir PD, Myovision,^21^ and KODAK) designed to reduce peripheral hyperopic defocus and introduce relative myopic defocus in the peripheral retina. The details for the LD and PD groups are presented in Table 1 and Supplementary Table S1.^21^^–^^23^ (3) The OK group: these OK lenses reshaped the corneal epithelium to induce peripheral myopic defocus^24^ (OK lens subtypes are detailed in Supplementary Table S2^25^^–^^27^). (4) The SV group: this was the control group, with single-vision spectacle lenses.

**Table 1.** Detailed Information on the Lenses in the LD and PD Groups

Group	Subtype	Brand and Manufacturer	*N*
LD	DIMS	MiyoSmart, HOYA corp, Japan	1050
	HAL	Stellest, essilor international, France	670
	IORC	SiWen, thondar, China	113
	Other type	N/A	79
PD	YAGE PD	Yage, See Vision optical Co., Ltd., China	633
	Shamir MC	Shamir myopia control proseries, Shamir optical industry Ltd., Israel	219
	MyoVision	MyoVision, carl zeiss AG, Germany	62
	KODAK	Kodak, Jiangsu Wanxin optical Co., Ltd. China	61

*N*, number of participants; N/A, not applicable; LD, lenslet-based design spectacle lenses; PD, peripheral defocus spectacle lenses; DIMS, defocus incorporated multiple segments; HAL, highly aspherical lenslet; IORC, individualized ocular refraction customization; Other type, six commercially available lenses, five based on spherical micro-lens arrays and one (ZEISS MyoCare with Cylindrical Annular Refractive Element technology) based on a micro-cylindrical array (see Supplementary Table S1).

**Table 2.** Baseline Characteristics of the Analytic Cohort

	Total (*N* = 5182)	LD (*N* = 1912)	PD (*N* = 975)	OK (*N* = 507)	SV (*N* = 1788)	*P* Value
Sex						
Male	2536 (48.94%)	979 (51.20%)	475 (48.72%)	231 (45.56%)	851 (47.60%)	0.056^a^
Female	2646 (51.06%)	933 (48.80%)	500 (51.28%)	276 (54.44%)	937 (52.40%)	
Age, y	10.84 (9.30 to 12.51)	10.39 (9.01 to 12.13)	11.20 (9.48 to 12.78)	11.17 (9.77 to 12.71)	11.02 (9.45 to 12.69)	<0.001^b^
6–11 y	3488 (67.31%)	1407 (73.59%)	610 (62.56%)	333 (65.68%)	1138 (63.65%)	<0.001^a^
12–16 y	1694 (32.69%)	505 (26.41%)	365 (37.44%)	174 (34.32%)	650 (36.35%)	
SER (D)	−2.00 (−3.00 to −1.25)	−2.00 (−3.00 to −1.38)	−2.00 (−3.00 to −1.31)	−2.25 (−3.50 to −1.50)	−1.94 (−2.88 to −1.25)	<0.001^b^
LM, *n*	3946 (76.15%)	1438 (75.21%)	753 (77.23%)	349 (68.84%)	1406 (78.64%)	<0.001^a^
MM, *n*	1236 (23.85%)	474 (24.79%)	222 (22.77%)	158 (31.16%)	382 (21.36%)	
AL, mm	24.46 (23.93 to 25.06)	24.43 (23.87 to 25.06)	24.52 (24.02 to 25.00)	24.57 (24.06 to 25.23)	24.40 (23.90 to 24.96)	<0.001^b^
AL, *n*	2801	1062	393	507	839	

AL, axial length; D, diopter; LD, lenslet-based design spectacle lenses; LM, low myopia (>−3.00 to ≤−0.50 D); mm, millimeter; MM, moderate myopia (≥−5.875 to ≤ −3.00 D); *n*, number of participants; OK, orthokeratology contact lenses; PD, peripheral defocus spectacle lenses; SER, spherical equivalent refraction; SV, single-vision spectacle lenses.

Continuous variables are presented as the median (interquartile range) because they were non-normally distributed (Shapiro–Wilk test, all *P* < 0.05). Categorical variables are presented as number (%).

a Chi-square test.

b Kruskal–Wallis.

### Data Categories and Follow-Up

Age stratification involved a median-split of participants into younger (6–11 years) and older (12–16 years) subgroups. The baseline SER categories were low myopia (LM), classified as >−3.00 to ≤−0.50 D, and moderate myopia (MM), classified as ≥−5.875 to ≤−3.00 D.^28^ Only AE was used as an outcome measure for the OK group (refraction omitted due to ongoing lens use), whereas AE and SER progression were assessed for the spectacle groups (LD, PD, and SV lenses). The follow-up duration was 12 ± 2 months.

### Statistical Analysis

The effectiveness of the myopia control was assessed by SER change and AE (right eye). Statistical analyses were conducted using SPSS software (version 26.0; IBM Corporation, Armonk, NY). The normality of continuous variables was tested with the Shapiro–Wilk test, and non-normally distributed variables were analyzed using the Kruskal–Wallis test for inter-group comparisons in descriptive statistics. Categorical variables were evaluated with a chi-square test. The agreement between the different refraction methods was assessed using Bland–Altman analysis. To adjust for potential confounding factors, PSM was used to balance baseline characteristics—including sex, age, baseline AL, and baseline SER—across groups. The PSM procedure and subsequent analyses were performed using the MatchIt package in R (version 4.3.2), with the caliper set to 0.1 via iterative trial-and-error to maximize sample size. Consistency across subtypes within the same lens category was assessed using 1-way analysis of variance (ANOVA). Post hoc multiple comparisons were subsequently performed using Duncan's multiple range test to identify subtypes exhibiting significant inconsistency. Comparisons of SER or AE alterations across groups were analyzed using multiple linear regression (MLR) models to adjust for age, sex, baseline AL, baseline SER, and the inconsistent subtype. SER and AL changes were standardized for between-group comparisons by converting observed follow-up changes to 12-month equivalents using the following formula:

$$\begin{array}{c} \Delta_{\text{12-month}}=\frac{\Delta_{\text{follow-up}}}{T_{\text{follow-up}}}\times 12 \end{array}$$

where Δ_follow-up_ denotes the observed SER and AL change during the follow-up period, and *T*_follow-up_ represents the follow-up duration in months.

Continuous data are presented as the mean ± standard deviation (SD) for normally distributed variables, or as median (interquartile range) otherwise. Categorical data are reported as frequencies and percentages (%). Significance was defined as *P* < 0.05 in all analyses.

## Results

### Baseline Characteristics

**Figure 1.**
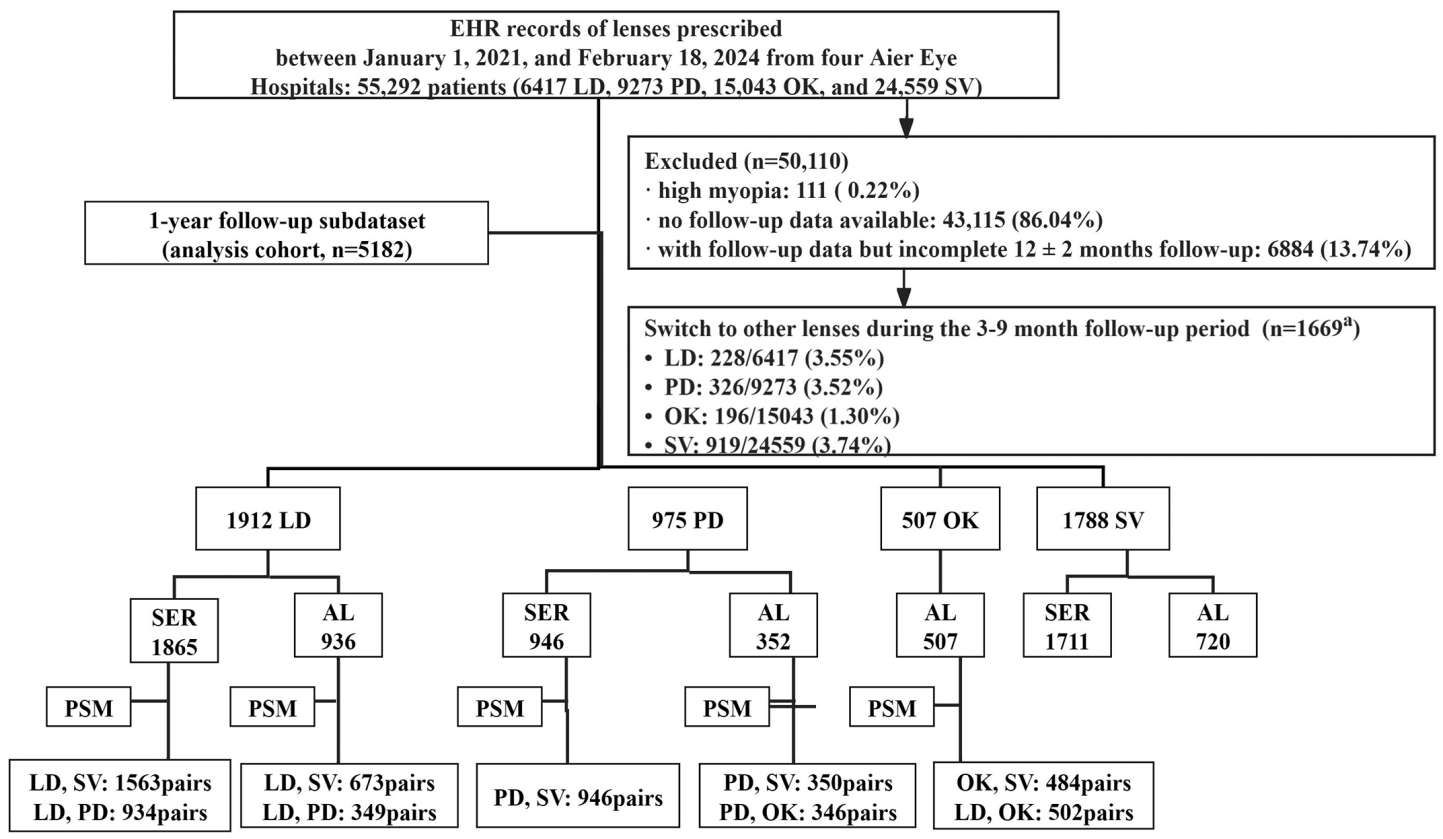
Flowchart of the data categories for the four groups of lenses (SER and AL results were not necessarily available for each individual patient). EHR, electronic health record; LD, lenslet-based design spectacle lenses; PD, peripheral defocus spectacle lenses; OK, orthokeratology contact lenses; SV, single-vision spectacle lenses; a: lens switches during 3 to 9 months (*n* = 1669) occurred in 6884 patients with incomplete 12 ± 2 months follow-up; SER, spherical equivalent refraction; AL, axial length; PSM, propensity score matching.

A total of 5182 participants were analyzed: 1912 in the LD group, 975 in the PD group, 507 in the OK group, and 1788 in the SV group. The baseline characteristics were as follows: mean age = 10.84 years (6–16 years); mean SER = −2.00 D (−0.50 to −5.875 D). Figure 1 shows a flowchart with the data categories for the four groups. The baseline characteristics of the analytic cohort are indicated in Table 2. Except for sex distribution (*P* > 0.05), the four groups differed significantly in age, SER categories, and baseline AL (*P* < 0.001). Table 3 demonstrates the baseline characteristics of each group following PSM (Supplementary Table S3 presents the data before and after PSM). After PSM was used to balance confounding factors across the cohorts, there were no significant differences in age, sex, baseline SER, or baseline AL across the lens groups (all *P* > 0.05).

**Table 3.** Baseline Characteristics of Each Group After PSM

After PSM (1-Y Follow-Up Sub-Dataset)									
SER	LD	SV	*P* Value	PD	SV	*P* Value	LD	PD	*P* Value
*N*	1563	1563		946	946		934	934	
Sex, % male	50.22	49.65	0.77	48.73	47.57	0.65	52.36	49.14	0.18
Age, y	10.75 ± 2.04	10.85 ± 1.98	0.16	11.12 ± 2.02	11.15 ± 2.04	0.74	11.11 ± 2.06	11.08 ± 2.01	0.78
SER, D	−2.29 ± 1.20	−2.24 ± 1.20	0.24	−2.29 ± 1.16	−2.29 ± 1.15	0.96	−2.32 ± 1.24	−2.30 ± 1.16	0.70
AL	LD	SV	*P* Value	PD	SV	*P* Value	OK	SV	*P* Value

*N*	673	673		350	350		484	484	
Sex, % male	50.67	49.33	0.66	49.71	52	0.6	45.45	44.83	0.9
Age, y	10.96 ± 2.05	11.03 ± 2.08	0.53	11.25 ± 2.10	11.15 ± 2.18	0.55	11.29 ± 2.01	11.29 ± 2.13	0.97
AL, mm	24.47 ± 0.84	24.46 ± 0.86	0.76	24.52 ± 0.79	24.55 ± 0.86	0.68	24.63 ± 0.85	24.63 ± 0.84	0.96
AL	LD	OK	*P* Value	LD	PD	*P* Value	PD	OK	*P* Value

*N*	502	502		349	349		346	346	
Sex, % male	47.21	46.02	0.75	52.15	49.86	0.6	49.13	46.82	0.59
Age, y	11.25 ± 2.04	11.26 ± 1.99	0.98	11.23 ± 2.14	11.22 ± 2.08	0.94	11.25 ± 2.08	11.27 ± 2.03	0.92
AL, mm	24.67 ± 0.89	24.66 ± 0.86	0.88	24.54 ± 0.9	24.53 ± 0.80	0.84	24.55 ± 0.78	24.60 ± 0.84	0.44

PSM, propensity score matching.

### SER Changes and AL Elongation in Different Groups

After standardizing the rate to 12-month values, subtype comparisons between the LD and PD group revealed no significant difference in AL elongation across the 4 subtypes in the PD group (*P* = 0.715; Supplementary Table S4). However, significant disparities across subtypes emerged in SER and AL progression in the LD group, as well as SER change in the PD group (*P* < 0.05). Post hoc analyses revealed that HAL and IORC subtypes in the LD group (which differed substantially from other subtypes) and the KODAK subtype in the PD group were adjustment factors. After multivariable adjustment for these variables, pairwise comparisons were made between the LD and PD groups. The significance of SER or AL comparisons among the LD, PD, OK, and SV groups remained unchanged after the “other” subtype of the LD group was removed (Supplementary Table S5). Nonsignificant differences were observed among subtypes within the OK group (*P* = 0.253; see Supplementary Table S2).

**Figure 2.**
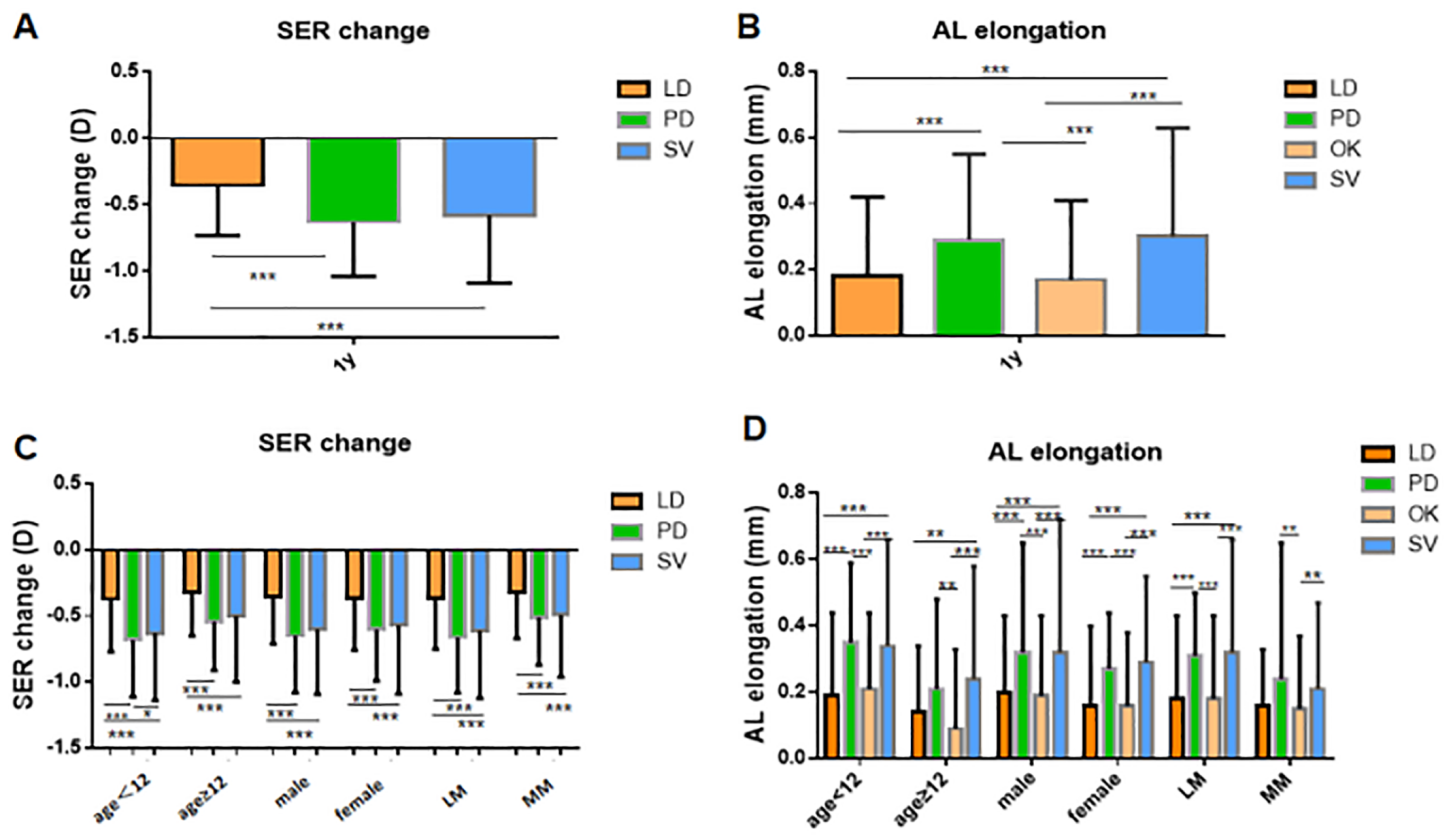
SER changes and AL elongation in different groups during the 1-year follow-up (MLR adjusted for age, sex, baseline AL, baseline SER and subtype, with monthly axial elongation rates standardized to 12-month equivalent values). (**A**) and (**B**) represent the SER changes and AL elongation in different groups; (**C**) and (**D**) represent the SER changes and AL elongation in subgroups by age, sex, and degrees of myopia; age <12 = 6 to 11 years; age ≥12 = 12 to 16 years; LM, low myopia (>−3.00 to ≤−0.50 D); MM, moderate myopia (≥−5.875 to ≤−3.00 D); *P < 0.05, **P < 0.01, ***P < 0.001.

SER changes and AL elongation in different groups during the 1-year follow-up are shown in Figures 2A and 2B (with detailed data in Supplementary Table S6).

Over 1 year, the LD group showed the smallest SER progression (mean = −0.35 ± 0.38 D), whereas the PD and SV groups demonstrated similar changes (−0.63 ± 0.41 D vs. −0.58 ± 0.51 D, *P* = 0.059; see Fig. 2A). Regarding AL, the mean elongation ranged from 0.17 ± 0.24 mm (in the OK group) to 0.30 ± 0.33 mm (in the SV group). No significant differences were found between the LD and OK groups (*P* = 0.738) or the PD and SV groups (*P* = 0.809; see Fig. 2B).

Following PSM, the LD group demonstrated superior myopia control efficacy compared with the PD and SV groups, as reflected in SER and AL progression, whereas the OK group showed AL elongation comparable to that observed in the LD group. These results were consistent with the MLR analysis.

### Treatment Efficacy

The effectiveness of various myopia control lenses relative to the SV group at 1 year is summarized in Table 4. Compared with the SV group, the LD group showed a greater decrease in SER, whereas the LD and OK groups demonstrated greater reductions in AE.

**Table 4.** Treatment Efficacy of Different Myopia Control Lenses at 1 Year

Changes of SER, D	LD/PD	SV	*P* Value	Treatment Efficacy, Mean ± SD
LD, *n* = 1865	−0.35 ± 0.38	−0.58 ± 0.51	<0.001	0.23 ± 0.09
PD, *n* = 946	−0.63 ± 0.41	−0.58 ± 0.51	0.059	−0.05 ± 0.09
AL elongation, mm	LD/PD/OK	SV	*P* Value	Treatment Efficacy, Mean ± SD

LD, *n* = 936	0.18 ± 0.24	0.30 ± 0.33	<0.001	−0.12 ± 0.06
PD, *n* = 352	0.29 ± 0.26	0.30 ± 0.33	0.809	−0.01 ± 0.06
OK, *n* = 507	0.17 ± 0.24	0.30 ± 0.33	<0.001	−0.13 ± 0.06

*n*, number of participants; SD, standard deviation.

MLR adjusted for age, sex, baseline AL, baseline SER, and subtype, with monthly axial elongation rates standardized to 12-month equivalent values.

### SER Changes and AL Elongation in Subgroups by Age, Sex, and Degrees of Myopia

In the subgroup analysis, the LD group demonstrated significantly slower SER progression than the PD and SV groups (all *P* < 0.05). In the younger age subgroup, the PD group exhibited significantly worse SER control than the SV group (−0.68 ± 0.43 D vs. −0.63 ± 0.51 D, *P* = 0.048). In the other subgroups, SER differences between the PD and SV groups were not significant (all *P* > 0.05). For AL elongation, there were no significant differences between the LD and OK groups or the PD and SV groups (all *P* > 0.05). In the MM subgroup, the LD group exhibited no significant differences from the SV and PD groups; the same finding was observed for the PD group in the older age subgroup (all *P* > 0.05; Figs. 2C, 2D, see Supplementary Table S6).

### Distribution of SER Progression and AL Elongation

**Figure 3.**
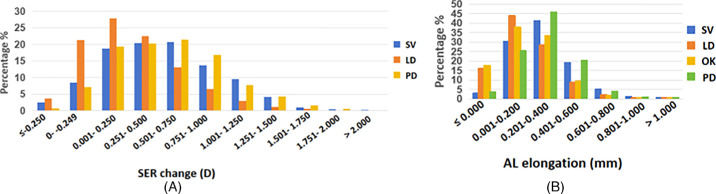
Distribution of SER progression and AL elongation across various lens types. (**A**) SER change distribution; (**B**) AL elongation distribution.

The 1-year distributions of SER and AL progression across the 4 lens types are shown in Table 5 and Figure. 3. The LD group showed favorable myopia control, with SER progression ≤0.50 D^14^ and AL elongation ≤0.20 mm.^17^

**Table 5.** Distribution of SER Progression and AL Elongation With Four Types of Lenses

Lens Group	Progression of SER ≤0.5 D	Elongation of AL ≤0.20 mm
SV	50.0% (855/1711)	33.5% (241/720)
LD	75.4% (1406/1865)	59.8% (560/936)
PD	47.5% (449/946)	30.4% (107/352)
OK	N/A	56.0% (284/507)

OK lens SER data unavailable due to continuous lens use.

### Adverse Events

During the 1-year follow-up period, 45 patients (8.88%, 45/507) in the OK group developed grade 1 corneal staining, and 5 patients (0.99%, 5/507) developed grade 2 corneal staining. No other adverse events were recorded. For mild corneal staining (grade 1), patients were permitted to continue using OK lenses and given recommendations to use preservative-free artificial tears and to adhere to strict follow-up. For patients with mild corneal staining (grade 2), epithelial-healing agents were administered, and strict adherence to proper lens handling, care procedures, and regular follow-up was emphasized. If corneal staining did not improve despite these measures, lens wear was discontinued, and management by a specialist was advised. Lens wear could be resumed only after complete corneal healing. During follow-up, nonsignificant local or systemic adverse reactions were observed in the spectacle lens group.

## Discussion

This RWS evaluated the 1-year myopia-control efficacy of LD, PD, and OK lenses compared with SV spectacle lenses in 5182 children and adolescents in Chongqing, China. Statistical analyses demonstrated that LD and OK lenses were comparable in slowing AL, outperforming PD and SV lenses. In contrast, PD lenses failed to reduce either SER progression or AL elongation compared with SV spectacle lenses. These findings were consistent across subgroups, stratified by age, sex, and baseline myopia severity (low or moderate).

The overall limited efficacy of PD spectacle lenses in controlling myopia progression is consistent with the findings of prior RCTs.^21^^,^^29^ However, subgroup analysis from an RCT-based study by Sankaridurg et al.^29^ suggested that PD spectacle lenses effectively slow myopia progression in the 6 to 12 year age group. This discrepancy may stem from the higher baseline SER in our cohort. Our findings indicate that LD spectacle lenses are generally more effective than conventional PD designs in slowing myopia progression. This difference can likely be attributed to the following mechanisms. The defocus effect of PD lenses depends on the wearer viewing the object through a specific area of the lens. The poor myopia control performance of PD spectacle lenses may be caused by dynamic misalignment between the visual axis and lens optical center during ocular movements. Furthermore, Kanda et al.^21^ hypothesized that the therapeutic impact of peripheral hyperopic defocus correction might not be adequately achieved with conventional spectacle lenses. However, the LD of LD lenses is associated with retinal image quality, which may affect their myopia‑control efficacy.^30^ In addition, LD lenses avoid peripheral light convergence, generating multiple point spread functions and low-contrast images that provide inhibitory optical stimuli for AL elongation, a mechanism consistent with peripheral retinal modulation of myopia.^31^ In summary, the difference in myopia control efficacy between LD and PD lenses may be due to the presence or absence of myopic defocus, the spatial distribution of defocused signals, and the resulting integrated differences in the bio-optical environment. Despite these findings, the precise mechanism by which myopia control is modulated remains incompletely understood and merits further research.

Studies comparing the efficacy of LD spectacle lenses and OK lenses in controlling AL elongation have yielded inconsistent results. For example, consistent AL control between LD and OK lenses was observed in RWSs by Yang et al.^17^ and Lee et al.^32^ over 1 year, with the former further demonstrating consistency across different lens brands. However, some RCTs^33^^,^^34^ demonstrated superior AL control with OK lenses over LD lenses in specific subpopulations, and a study by Lai et al.^8^ revealed better AL elongation control in the LD group. These discrepancies may stem from heterogeneity in study population definitions and subgroup classification criteria for low, moderate, and high myopia. Lu et al.^33^ enrolled participants aged 7–14 years and categorized LM as −1.50 D ≤ SER ≤ −0.50 D with a SER range of −0.5 D to −5.0 D, whereas Lai et al.^8^ included participants aged 7 to 13 years with SER of −0.25 D to −3.0 D. In contrast, our study encompassed a broader age range (6–16 years) and refractive span (SER of −0.50 D to −5.875 D). Second, the wearing mode differs significantly between OK lenses and spectacle lenses. Spectacle lenses are easy to use, which may naturally lead to better adherence. In contrast, OK lenses require strict compliance, such that the duration of nightly wear can significantly influence therapeutic efficacy.^35^ However, Hung et al.^36^ found that 10.76% of parents were unaware of this nightly wear requirement, highlighting the need for clearer patient education and professional support. Third, differences between RCT and RWS methodologies may lead to variations in outcomes, as RCTs often fail to reflect real-world scenarios due to strict patient selection, a focus on compliant and responsive populations, and specialized clinical settings distinct from general practice environments.^37^ Real-world data enables clinicians to appraise the translational value of RCT results in heterogeneous patient populations.^38^ Consequently, by reflecting authentic clinical scenarios, this study offers clinically relevant findings.

Our study involved a 1-year follow-up wherein it was found that the average changes in SER and AL in the LD group were −0.35 D and 0.18 mm, respectively, showing superior efficacy (by −0.23 D and 0.12 mm, respectively) over the SV control group. These findings align with previously acquired RWS data, which reported 1-year SER progression of −0.22 D to −0.50 D^10^^,^^14^^,^^17^ and AL elongation of 0.16 to 0.20 mm^10^^,^^17^ for LD lenses. The observed treatment benefits of LD lenses over SV lenses (SER reduction = −0.27 D^14^ and AL inhibition = 0.11–0.23 mm^39^) is consistent with the variability typical of RWE datasets. Lan et al.^14^ reported reduced SER control efficacy with DIMS compared with our findings, which may be attributable to their inclusion of patients with high myopia. However, we included OK lenses for comparison, and high myopia participants were excluded from the study population to ensure comparability across lens groups. A notable methodological limitation of Lan's study is its omission of AL measurements, which precludes a comprehensive assessment of myopia control efficacy. Furthermore, RCTs evaluating LD spectacle lens subtypes have demonstrated 1-year progression ranges from −0.16 D to −0.48 D in SER,^39^^,^^40^ and 0.12 to 0.30 mm in AL.^33^^,^^40^^,^^41^ The substantial variability across RCTs likely stems from population heterogeneity in baseline characteristics, highlighting the necessity for standardized inclusion criteria in myopia control research.

In the stratified analysis, regarding participants with moderate myopia, the AL elongation for LD, PD, SV, and OK lenses was 0.16, 0.24, 0.21, and 0.15 mm, respectively. Similarly, for the population aged 12 to 16 years, the AE values for the 4 lens types were 0.14, 0.21, 0.24, and 0.09 mm. Compared with PD and SV lenses, LD lenses exhibited slower AE. However, this difference was not significant (*P* > 0.05), possibly due to there being a smaller subgroup sample size after stratification, which diminished statistical power. Future studies should aim to increase the sample size to address this limitation. In other stratified analyses, the OK and LD lenses consistently demonstrated superior myopia control efficacy, as evidenced by significantly slower SER and AL progression relative to PD and SV lenses.

For OK lenses, our observed AL elongation (0.17 mm/year) aligns with the findings of a previous RWS (0.18–0.19 mm/year)^17^ and RCTs (0.15–0.22 mm/year).^33^^,^^41^^,^^42^ However, Yang et al.^17^ and Skidmore et al.^11^ did not include an SV control group; thus, no direct comparison of the true clinical effectiveness of an AE control with OK lenses is possible. Additional real-world research is needed for verification. We acknowledge the inherent baseline disparities between groups during the initial comparisons, particularly regarding age differences between OK lens and spectacle lens users. However, PSM successfully balanced these covariates (all *P* > 0.05).^16^ Real-world observational research inevitably faces confounding factors that may compromise causal interpretations. To address this challenge, PSM—a robust statistical approach widely used to mitigate selection bias—was implemented to improve validity by achieving covariate equilibrium between treatment and control groups. This method aligns with established practices in real-world research on myopia control.^14^

This study included different types of myopia-control lenses. OK lens subtypes showed consistent AL control across brands, aligning with prior studies assessing different designs.^43^^,^^44^ In contrast, the comparative efficacy of LD lens subtypes in myopia control remains inconclusive. For example, Guo et al.^45^ showed that HAL outperformed DIMS in controlling SER progression (−0.63 D) and AL elongation (0.27 mm) over 1 year. However, the progression observed in DIMS (−0.63 D SER, 0.27 mm AL) was higher than that reported in other 1-year studies of DIMS, including by Wen et al.^6^ (−0.50 D SER) and a Hong Kong cohort^18^ (−0.17 D SER, 0.11 mm AL). Conversely, evidence from RCTs^40^ indicates comparable efficacy between HAL and DIMS subtypes in modulating both SER and AL progression. Limited comparative data exist for IORC lenses relative to other multifocal designs. As demonstrated in previous research, there are differences in specific optical parameters, such as the add power and distribution density of microlenses among different LD lens designs.^30^ However, there is currently insufficient evidence to indicate that switching between different lens designs is related to the effectiveness of myopia control. In a study by Su et al.,^46^ no significant difference in myopia control efficacy was observed between wearing one lens design continuously over 2 years and switching to a different lens design each year. The objective of this study was to assess the relative efficacy of major optical strategies for myopia control in real-world settings. It was not intended to compare the subtypes within any specific lens category. Therefore, to control for this potential confounding factor in the pooled analysis, lens subtype was included as a covariate in all MLR models, thereby statistically adjusting for the influence of inter‑subtype differences on the estimation of overall effects. However, merging different brands for analysis may not fully reveal the optimal performance of specific brands in particular populations. To better inform and refine clinical strategies, prospective, large-scale comparative studies are warranted.

Over the 1-year follow-up period, corneal staining (grade 1 = 8.88% and grade 2 = 0.99%) was observed with the OK lens, and no clinically significant local or systemic adverse reactions was reported with the spectacles lens. Our observed incidence of adverse events across 1 year with OK lenses was lower than that indicated in previous RWSs (Yang et al.^17^ and Song et al.^47^; grade 1 = 11.3–15.1% and grade 2 = 1.9–3.8%). Notably, none of these studies identified any serious complications associated with OK lenses. This study is consistent with the findings of Yang et al.^17^ and Gupta et al.,^30^ and no adverse reactions related to the lenses were observed during the follow-up period. However, this study did not specifically assess lens adaptation. Fan et al.^10^ and Lam et al.^48^ found that LD lenses could be adapted within days without affecting daily life.

This study demonstrates several strengths through its comprehensive design. First, it provides real-world clinical evidence on the efficacy of diverse myopia control lenses, leveraging multicenter data from four major ophthalmic hospitals in Chongqing with a substantial sample size. Second, the protocol incorporated multidimensional biometric parameters, such as AL and SER, enabling stratified analyses across varying myopia severities, age groups, and sexes. Third, applying the PSM methodology and the MLR model effectively balanced baseline confounders inherent to observational studies, thereby improving the validity of comparative outcomes.

This study also has certain limitations. First, it is an RWS, and lens selection is influenced by complex factors, including clinical preferences, patient baseline characteristics, and socioeconomic determinants. To address this, we rigorously controlled for known confounding factors through various statistical methods such as PSM, multivariate adjustment, and extensive subgroup analysis (stratified by age, sex, and baseline degree). The larger sample size and multicenter design may have substantially minimized potential biases.^49^ Collectively, these methodological approaches support the robustness of the primary conclusions. Despite our efforts, residual confounding might remain, as genetic and environmental influences and wear compliance were not systematically analyzed.^36^^,^^50^ Thus, our findings should be considered as comparative effectiveness estimates of three myopia control interventions in real-world settings, with causal inference warranting further validation in future RCTs. Second, this retrospective study was unable to confirm whether the enrolled participants had sufficient washout periods prior to enrollment. Although we identified patients who were fitted with only one lens type during the study period through data screening, this limitation may have introduced residual selection bias and confounding from pre-existing treatment effects. Notably, the potential limitations of each lens group compared in this study are consistent. The comparative effectiveness of myopia control strategies demonstrated in this RWS remains clinically significant. Third, treatment discontinuation or switching due to poor response or preference may have introduced selective persistence bias, potentially biasing the study population toward those with better outcomes. To investigate this potential bias, we analyzed the lens switching rates. We observed that OK lens wearers exhibited the lowest lens switching rate (1.30%), whereas SV spectacle lens wearers exhibited the highest (3.74%). This disparity may be attributable to the superior adherence and user satisfaction associated with the OK lens-wearing regimen. The higher lens-switching rate associated with SV spectacle lenses (the control group) potentially reflects proactive clinical decision making by parents pursuing more effective intervention in response to rapid myopia progression. The observed inverse relationship between efficacy and treatment behavior constitutes an important real-world finding. Ultimately, the net clinical effect emerges from the combined influence of optical strategy and patient behavior. To better quantify this bias, future prospective RWSs should systematically and prospectively record the precise reasons for lens discontinuation or switching. Finally, this study was limited to evaluating lens adaptation or visual quality. These aspects should be addressed in future research.

## Conclusions

In this RWS with a 1-year follow-up period, LD and OK lenses demonstrated superior myopia control efficacy compared with PD and SV lenses. LD and OK lenses indicated consistent and comparable effectiveness in slowing AL elongation across age, sex, and low-to-moderate myopia subgroups. In contrast, PD lenses exhibited limited effectiveness in controlling both SER progression and AL elongation. These results highlight that LD and OK lenses are more reliable clinical options for myopia management in diverse pediatric populations. Future studies should prioritize longer follow-up periods, brand-specific analyses, and integration of genetic and environmental factors to further refine myopia management strategies.

## Supplementary Material

Supplement 1

Supplement 2
