# Supplementary material for: Comparative Effectiveness of Three Myopia Control Lenses: A Multicenter, Real-World Study of 5182 Adolescents and Children in Chongqing, China
Source: Transl Vis Sci Technol. 2026 May 27;15(5):22. doi: 10.1167/tvst.15.5.22 (PMC13221893; doi:10.1167/tvst.15.5.22)
Supplement: Supplement 1 [file tvst-15-5-22_s001.pdf]

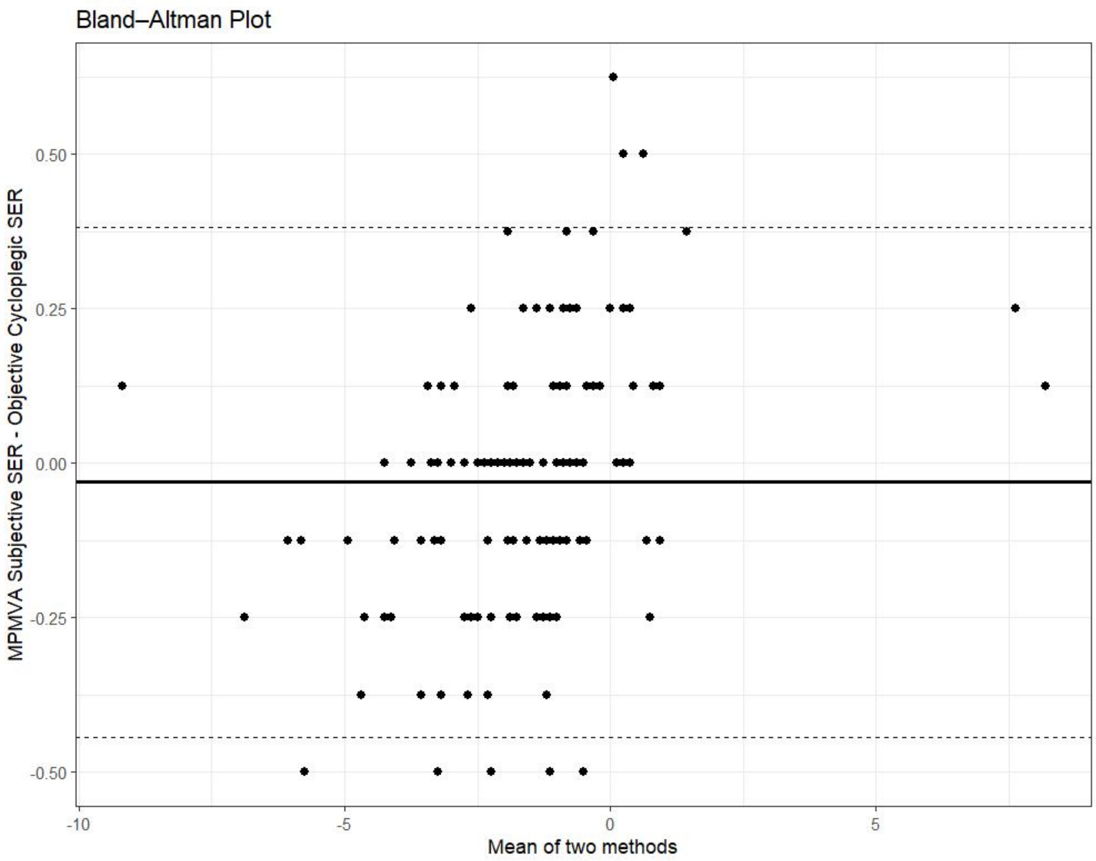

Supplementary Figure 1: Bland-Altman analysis for the agreement of Spherical Equivalent Refraction (SER) between manifest subjective refraction (MPMVA) and objective refraction under cycloplegic : number=155 eyes (80 patients) ; Bias = -0.031, 95%LoA: -0.444 to 0.383; ICC=0.994, 95% CI: 0.992-0.996.

Note: The analysis was based on a randomly selected subset of 155 eyes from 80 participants to validate the consistency of the subjective method. The solid line represents the mean difference, and the dashed lines represent the 95% limits of agreement.
