# Supplementary material for: Comparative Effectiveness of Three Myopia Control Lenses: A Multicenter, Real-World Study of 5182 Adolescents and Children in Chongqing, China
Source: Transl Vis Sci Technol. 2026 May 27;15(5):22. doi: 10.1167/tvst.15.5.22 (PMC13221893; doi:10.1167/tvst.15.5.22)
Supplement: Supplement 2 [file tvst-15-5-22_s002.pdf]

Supplement Table 1. Detailed information of LD and PD lens subtypes

| Group | Subtype | N | Brand and manufacturer | Key design Features | References/ Source |
| --- | --- | --- | --- | --- | --- |
| LD | DIMS | 1050 | MiyoSmart, HOYA Corp, Japan | Multiple defocus segments around central zone (396 microlenses are arranged across a 32 mm diameter area surrounding the optical zone) | https://www.hoyavision.com/cn/vision-products/MiYOSMART/ |
| | HAL | 670 | Stellest, Essilor International, France | Thousands of aspherical lenslets on surface | https://www.essilorchina.com/stellest |
| | IORC | 113 | SiWen, Thondar, China | Custom-mapped correction per patient | https://web.thondar.cn/ |
| | YAGE LD | 33 | AnXingYue, See Vision Optics Co., Ltd., China | A 10 mm central zone is surrounded by 485 microlenses | N/A[a] |
| | YiBaiFen | 32 | YiBaiFen, Jiangsu Wanxin Optical Co., Ltd., China | 480 microlenses are arranged in a continuous dot matrix | N/A[a] |
| | DSDO | 6 | AURA,Zhuhai Fitlens Ltd, China | Diversified segmental defocus optimization (a central optical zone (9.5 mm in diameter) and 256 microlenses in the peripheral field) | [22] |
| | Shamir LD | 4 | Shamir LD, Shamir Optical Industry Ltd., Israel | Dual-surface compound design of 8 annular multi-point microlenses rings combined with hyperopic defocus | N/A[b] |
| | CARE | 3 | MyoCare, Zeiss Vision Care, Aalen, Germany | Micro-cylinder array | [23] |
| | Kulifishi | 1 | Kulifishi, Suzhou Champion Fine Optical Co., Ltd., China | Compound multi-point microlenses in a tapered spiral configuration | Chinese patent number: CN202121094359.1 |
| PD | YAGE PD | 633 | Yage, See Vision Optics Co., Ltd., China | asymmetric configuration, peripheral aspherical power distribution (+2.5D) | N/A[a] |
| | Shamir MC | 219 | Shamir myopia control pro series, Shamir Optical Industry Ltd., Israel | asymmetric configuration, peripheral aspherical power distribution (+1.5D) | https://shamir.com |

| | | | | |
|---|---|---|---|---|
| MyoVision | 62 | MyoVision, Carl Zeiss AG, Germany | asymmetric configuration, peripheral aspherical power distribution (+1.9D) | [21] |
| KODAK | 61 | Kodak, Jiangsu Wanxin Optical Co., Ltd., China | asymmetric configuration, peripheral aspherical power distribution | N/A[a] |

N: number of participants; LD: lenslet-based design spectacle lenses; PD: peripheral defocus spectacle lenses; DIMS: Defocus Incorporated Multiple Segments; HAL: Highly Aspherical Lenslet; IORC: Individualized ocular refraction customization; DSDO: diversified segmental defocus optimization; CARE: cylindrical annular refractive element; N/A: Not applicable; a: obtained from offline stores; no official links or literature available; b: this subtype has been discontinued.

Supplementary Table 2. Detailed Information of OK Lens Subtypes

| Group | Subtype | N | Brand and manufacturer | Key design Features | References/ Source | *P* |
|---|---|---|---|---|---|---|
| OK | iBright | 215 | iBright, Eyebright Medical Technology (Beijing) Co., Ltd., China | VST design | http://www.ebmedical.com/proc/light/pnt/2020-06-02/22.html | *P1*=0.253 *P2*=0.717 |
| | Alpha | 123 | Alpha, Alpha Corporation, Japan | VST design | [25] | |
| | Lucid | 48 | Lucid, Lucid Korea Co. Ltd, Korea | VST design | [27] | |
| | Euclid | 52 | Euclid, Euclid Systems Corporation, USA | VST design | [25] | |
| | DreamVision | 37 | DreamVision, Polymer Technology Corp., USA | VST design | [26] | |
| | Dreamlite | 17 | Dreamlite, Procornea Ltd, The Netherlands | CRT design | [27] | |
| | CRT | 12 | CRT, Paragon Vision | CRT design | [25] | |

| | | | | |
|---|---|---|---|---|
| | | Sciences, Inc., USA | | |
| Hiline | 3 | Hiline, Nanpeng Hiline Inc., China | VST lenses | [25] |

VST: Vision shaping treatment; CRT: corneal reshaping therapy; N: number of participants; *P1*: *P*-value for comparisons among OK lens subtypes (ANOVA); *P2: P*-value for the comparison between CRT design and VST design (independent samples t-test).

Supplement Table 3. Baseline characteristics of each group before and after PSM

| | Before PSM | | | After PSM | | |
|---|---|---|---|---|---|---|
| **SER** | LD | SV | *P* | LD | SV | *P* |
| Number | 1865 | 1711 | | 1563 | 1563 | |
| Sex(male%) | 51.15 | 47.98 | 0.06 | 50.22 | 49.65 | 0.77 |
| Age(year) | 10.57 ± 2.04 | 11.09 ± 2.07 | 0.00 | 10.75 ± 2.04 | 10.85 ± 1.98 | 0.16 |
| SER(D) | -2.34 ± 1.22 | -2.17 ± 1.19 | 0.00 | -2.29 ± 1.2 | -2.24 ± 1.2 | 0.24 |
| **SER** | PD | SV | *P* | PD | SV | *P* |
| Number | 946 | 1711 | | 946 | 946 | |
| Sex(male%) | 48.73 | 47.98 | 0.74 | 48.73 | 47.57 | 0.65 |
| Age(year) | 11.12 ± 2.02 | 11.09 ± 2.07 | 0.72 | 11.12 ± 2.02 | 11.15 ± 2.04 | 0.74 |
| SER(D) | -2.29 ± 1.16 | -2.17 ± 1.19 | 0.01 | -2.29 ± 1.16 | -2.29 ± 1.15 | 0.96 |
| **SER** | LD | PD | *P* | LD | PD | *P* |
| Number | 1865 | 946 | | 934 | 934 | |
| Sex(male%) | 51.15 | 48.73 | 0.24 | 52.36 | 49.14 | 0.18 |
| Age(year) | 10.57 ± 2.04 | 11.12 ± 2.02 | 0.00 | 11.11 ± 2.06 | 11.08 ± 2.01 | 0.78 |
| SER(D) | -2.34 ± 1.22 | -2.29 ± 1.16 | 0.32 | -2.32 ± 1.24 | -2.3 ± 1.16 | 0.70 |
| **AL** | LD | SV | *P* | LD | SV | *P* |
| Number | 936 | 720 | | 673 | 673 | |

| | | | | | | |
|---|---|---|---|---|---|---|
| Sex(male%) | 51.71 | 48.89 | 0.28 | 50.67 | 49.33 | 0.66 |
| Age(year) | 10.73 ± 2.07 | 11.2 ± 2.12 | 0.00 | 10.96 ± 2.05 | 11.03 ± 2.08 | 0.53 |
| AL (mm) | 24.5 ± 0.86 | 24.44 ± 0.85 | 0.12 | 24.47 ± 0.84 | 24.46 ± 0.86 | 0.76 |
| **AL** | PD | SV | *P* | PD | SV | *P* |
| Number | 352 | 720 | | 350 | 350 | |
| Sex(male%) | 51.11 | 48.89 | 0.78 | 49.71 | 50.29 | 0.60 |
| Age(year) | 11.25 ± 2.1 | 11.2 ± 2.12 | 0.71 | 11.25 ± 2.1 | 11.15 ± 2.18 | 0.55 |
| AL (mm) | 24.53 ± 0.79 | 24.44 ± 0.85 | 0.08 | 24.52 ± 0.79 | 24.55 ± 0.86 | 0.68 |
| **AL** | OK | SV | *P* | OK | SV | *P* |
| Number | 507 | 720 | | 484 | 484 | |
| Sex(male%) | 45.56 | 48.89 | 0.28 | 45.45 | 44.83 | 0.90 |
| Age(year) | 11.3 ± 2.02 | 11.2 ± 2.12 | 0.40 | 11.29 ± 2.01 | 11.29 ± 2.13 | 0.97 |
| AL (mm) | 24.67 ± 0.86 | 24.44 ± 0.85 | 0.00 | 24.63 ± 0.85 | 24.63 ± 0.84 | 0.96 |
| **AL** | LD | OK | *P* | LD | OK | *P* |
| Number | 936 | 507 | | 502 | 502 | |
| Sex(male%) | 51.71 | 45.56 | 0.03 | 47.21 | 46.02 | 0.75 |
| Age(year) | 10.73 ± 2.07 | 11.3 ± 2.02 | 0.00 | 11.25 ± 2.04 | 11.26 ± 1.99 | 0.98 |
| AL (mm) | 24.5 ± 0.86 | 24.67 ± 0.86 | 0.00 | 24.67 ± 0.89 | 24.66 ± 0.86 | 0.88 |
| **AL** | LD | PD | *P* | LD | PD | *P* |
| Number | 936 | 352 | | 349 | 349 | |
| Sex(male%) | 51.71 | 48.29 | 0.63 | 52.15 | 49.86 | 0.60 |
| Age(year) | 10.73 ± 2.07 | 11.25 ± 2.1 | 0.00 | 11.23 ± 2.14 | 11.22 ± 2.08 | 0.94 |
| AL (mm) | 24.5 ± 0.86 | 24.53 ± 0.79 | 0.61 | 24.54 ± 0.90 | 24.53 ± 0.80 | 0.84 |
| **AL** | PD | OK | *P* | PD | OK | *P* |
| Number | 352 | 507 | | 346 | 346 | |
| Sex(male%) | 54.44 | 45.56 | 0.23 | 49.13 | 46.82 | 0.59 |

| | | | | | | |
|---|---|---|---|---|---|---|
| Age(year) | 11.25 ± 2.1 | 11.3 ± 2.02 | 0.72 | 11.25 ± 2.08 | 11.27 ± 2.03 | 0.92 |
| AL (mm) | 24.53 ± 0.79 | 24.67 ± 0.86 | 0.00 | 24.55 ± 0.78 | 24.6 ± 0.84 | 0.44 |

PSM: propensity score matching.

Supplement Table 4. Comparison of SER（D）Changes and AL (mm) Elongation Among Subgroups Within LD and PD group during 1-year follow-up

| LD | DIMS | HAL | IORC | Other Subgroup | *P* | Post Hoc |
|---|---|---|---|---|---|---|
| N | 1050 | 670 | 113 | 79 | | |
| Age (years) | 10.48±2.04 | 10.69±1.99 | 10.67±2.05 | 10.63±2.30 | | |
| Changes of SER (n) | -0.38±0.38 (n=1031) | -0.30±0.39 (n=651) | -0.51±0.38 (n=107) | -0.39±0.45 (n=76） | 0.000 | *P1*=0.062 *P2*=0.804 |
| AL elongation (n) | 0.20±0.19 (n=476) | 0.13±0.289 (n=316) | 0.36±1.20 (n=84) | 0.21±0.18 (n=61) | 0.000 | *P3*=0.189 |
| PD | YAGE PD | SHAMIR MC | MYOVISION | KODAK | | |
| N | 633 | 219 | 62 | 61 | | |
| Age (years) | 11.14±1.96 | 10.89±2.08 | 11.31±2.08 | 11.52±2.26 | | |
| Changes of SER (n) | -0.61±0.41 (n=615) | -0.67±0.41 (n=211) | -0.71±0.39 (n=61) | -0.80±0.48 (n=59) | 0.002 | *P4*=0.117 *P5*=0.096 |
| AL elongation (n) | 0.31±0.25 (n=190) | 0.28±0.33 (n=120) | 0.30±0.18 (n=20) | 0.29±0.18 (n=2) | 0.715 | |

N(n): number of participants; *P*: One-way ANOVA; Post Hoc comparisons: *P1*: DIMS vs HAL(SER); *P2*: DIMS vs other subgroup (SER); *P3*: DIMS vs HAL vs other subgroup (AL); *P4*: MYOVISION vs KODAK(SER); *P5*: MYOVISION vs SHAMIR PD vs YAGE PD(SER).

Supplement Table 5. SER changes and AL elongation in different groups at one year (excluding the other subgroup of LD group)

| SER | LD | PD | SV | *P1* | *P2* | *P3* |
|---|---|---|---|---|---|---|
| total | -0.35±0.38 | -0.63±0.41 | -0.58±0.51 | 0.000 | 0.058 | 0.000 |
| （n） | （1789） | （946） | （1711） | | | |
| age<12 | -0.37±0.40 | -0.68±0.43 | -0.63±0.51 | 0.000 | 0.047 | 0.000 |
| （n） | （1321） | （593） | （1078） | | | |
| age≥12 | -0.32±0.33 | -0.54±0.37 | -0.50±0.50 | 0.000 | 0.625 | 0.000 |
| （n） | （468） | （353） | （633） | | | |
| male | -0.35±0.36 | -0.65±0.43 | -0.60±0.49 | 0.000 | 0.185 | 0.000 |
| （n） | （911） | （461） | （821） | | | |
| female | -0.36±0.40 | -0.60±0.39 | -0.57±0.52 | 0.000 | 0.169 | 0.000 |
| （n） | （878） | （485） | （890） | | | |
| LM | -0.36±0.39 | -0.66±0.42 | -0.61±0.51 | 0.000 | 0.137 | 0.000 |
| （n） | （1333） | （728） | （1344） | | | |
| MM | -0.32±0.35 | -0.51±0.36 | -0.49±0.47 | 0.002 | 0.108 | 0.000 |
| （n） | （456） | （218） | （367） | | | |

| AL | LD | PD | OK | SV | *P1* | *P2* | *P3* | *P4* | *P5* | *P6* |
|---|---|---|---|---|---|---|---|---|---|---|
| total | 0.18±0.24 | 0.29±0.26 | 0.17±0.24 | 0.30±0.33 | 0.000 | 0.812 | 0.000 | 0.000 | 0.708 | 0.000 |
| （n） | （875） | （352） | （507） | （720） | | | | | | |
| age<12 | 0.19±0.25 | 0.35±0.24 | 0.21±0.23 | 0.34±0.32 | 0.000 | 0.500 | 0.000 | 0.000 | 0.291 | 0.000 |
| （n） | （621） | （209） | （333） | （440） | | | | | | |
| age≥12 | 0.14±0.20 | 0.21±0.27 | 0.09±0.24 | 0.24±0.34 | 0.002 | 0.250 | 0.080 | 0.000 | 0.336 | 0.005 |
| （n） | （254） | （143） | （174） | （280） | | | | | | |
| male | 0.2±0.23 | 0.32±0.33 | 0.19±0.26 | 0.32±0.40 | 0.000 | 0.996 | 0.000 | 0.000 | 0.572 | 0.000 |
| （n） | （449） | （176） | （231） | （352） | | | | | | |
| female | 0.16±0.24 | 0.27±0.17 | 0.16±0.22 | 0.29±0.26 | 0.000 | 0.646 | 0.000 | 0.000 | 0.851 | 0.000 |

| | | | | | | | | | | |
|---|---|---|---|---|---|---|---|---|---|---|
| （n） | （426） | （176） | （276） | （368） | | | | | | |
| LM | 0.18±0.25 | 0.31±0.19 | 0.18±0.25 | 0.32±0.34 | 0.000 | 0.819 | 0.000 | 0.000 | 0.191 | 0.000 |
| （n） | （657） | （267） | （349） | （587） | | | | | | |
| MM | 0.16±0.17 | 0.24±0.41 | 0.15±0.22 | 0.21±0.26 | 0.163 | 0.544 | 0.069 | 0.002 | 0.091 | 0.001 |
| （n） | （218） | （85） | （158） | （133） | | | | | | |

n: number of participants; MLR adjusted for age, gender, baseline AL, SER, subgroups, with monthly axial elongation rates standardized to 12-month equivalent values; *P1*: LD vs SV; *P2*: PD vs SV; *P3*: LD vs PD; *P4*: OK vs SV; *P5*: LD vs OK; *P6*: PD vs OK

Supplement Table 6. SER changes and AL elongation in different groups at one year

| SER | LD | PD | SV | *P1* | *P2* | *P3* | | | | |
|---|---|---|---|---|---|---|---|---|---|---|
| total | -0.35±0.38 | -0.63±0.41 | -0.58±0.51 | 0.000 | 0.059 | 0.000 | | | | |
| （n） | （1865） | （946） | （1711） | | | | | | | |
| age<12 | -0.37±0.40 | -0.68±0.43 | -0.63±0.51 | 0.000 | 0.048 | 0.000 | | | | |
| （n） | （1374） | （593） | （1078） | | | | | | | |
| age≥12 | -0.32±0.33 | -0.54±0.37 | -0.50±0.50 | 0.000 | 0.633 | 0.000 | | | | |
| （n） | （491） | （353） | （633） | | | | | | | |
| male | -0.35±0.36 | -0.65±0.43 | -0.60±0.49 | 0.000 | 0.180 | 0.000 | | | | |
| （n） | （954） | （461） | （821） | | | | | | | |
| female | -0.36±0.40 | -0.60±0.39 | -0.57±0.52 | 0.000 | 0.173 | 0.000 | | | | |
| （n） | （911） | （485） | （890） | | | | | | | |
| LM | -0.36±0.39 | -0.66±0.42 | -0.61±0.51 | 0.000 | 0.139 | 0.000 | | | | |
| （n） | （1400） | （728） | （1344） | | | | | | | |
| MM | -0.32±0.35 | -0.51±0.36 | -0.49±0.47 | 0.002 | 0.106 | 0.000 | | | | |
| （n） | （465） | （218） | （367） | | | | | | | |
| AL | LD | PD | OK | SV | *P1* | *P2* | *P3* | *P4* | *P5* | *P6* |

| | | | | | | | | | | |
|---|---|---|---|---|---|---|---|---|---|---|
| total | 0.18±0.24 | 0.29±0.26 | 0.17±0.24 | 0.30±0.33 | 0.000 | 0.809 | 0.000 | 0.000 | 0.738 | 0.000 |
| （n） | （936） | （352） | （507） | （720） | | | | | | |
| age<12 | 0.19±0.25 | 0.35±0.24 | 0.21±0.23 | 0.34±0.32 | 0.000 | 0.498 | 0.000 | 0.000 | 0.263 | 0.000 |
| （n） | （660） | （209） | （333） | （440） | | | | | | |
| age≥12 | 0.14±0.20 | 0.21±0.27 | 0.09±0.24 | 0.24±0.34 | 0.001 | 0.244 | 0.068 | 0.000 | 0.293 | 0.004 |
| （n） | （276） | （143） | （174） | （280） | | | | | | |
| male | 0.2±0.23 | 0.32±0.33 | 0.19±0.26 | 0.32±0.40 | 0.000 | 0.990 | 0.000 | 0.000 | 0.613 | 0.000 |
| （n） | （484） | （176） | （231） | （352） | | | | | | |
| female | 0.16±0.24 | 0.27±0.17 | 0.16±0.22 | 0.29±0.26 | 0.000 | 0.644 | 0.000 | 0.000 | 0.891 | 0.000 |
| （n） | （452） | （176） | （276） | （368） | | | | | | |
| LM | 0.18±0.25 | 0.31±0.19 | 0.18±0.25 | 0.32±0.34 | 0.000 | 0.815 | 0.000 | 0.000 | 0.215 | 0.000 |
| （n） | （709） | （267） | （349） | （587） | | | | | | |
| MM | 0.16±0.17 | 0.24±0.41 | 0.15±0.22 | 0.21±0.26 | 0.148 | 0.541 | 0.062 | 0.002 | 0.088 | 0.001 |
| （n） | （227） | （85） | （158） | （133） | | | | | | |

n: number of participants; MLR adjusted for age, gender, baseline AL, SER, subgroups, with monthly axial elongation rates standardized to 12-month equivalent values; *P1*: LD vs SV; *P2*: PD vs SV; *P3*: LD vs PD; *P4*: OK vs SV; *P5*: LD vs OK; *P6*: PD vs OK
